# The Stability and Formation of Native Proteins from Unfolded Monomers Is Increased through Interactions with Unrelated Proteins

**DOI:** 10.1371/journal.pone.0000497

**Published:** 2007-06-06

**Authors:** Claudia Rodríguez-Almazán, Francisco J. Torner, Miguel Costas, Ruy Pérez-Montfort, Marieta Tuena de Gómez-Puyou, Armando Gómez Puyou

**Affiliations:** 1 Departamento de Bioquímica, Instituto de Fisiología Celular, Universidad Nacional Autónoma de México, Mexico City, Mexico; 2 Laboratorio de Biofisicoquímica, Departamento de Fisicoquímica, Facultad de Química, Universidad Nacional Autónoma de México, Mexico City, Mexico; Baylor College of Medicine, United States of America

## Abstract

The intracellular concentration of protein may be as high as 400 mg per ml; thus it seems inevitable that within the cell, numerous protein-protein contacts are constantly occurring. A basic biochemical principle states that the equilibrium of an association reaction can be shifted by ligand binding. This indicates that if within the cell many protein-protein interactions are indeed taking place, some fundamental characteristics of proteins would necessarily differ from those observed in traditional biochemical systems. Accordingly, we measured the effect of eight different proteins on the formation of homodimeric triosephosphate isomerase from *Trypanosoma brucei* (TbTIM) from guanidinium chloride unfolded monomers. The eight proteins at concentrations of micrograms per ml induced an important increase on active dimer formation. Studies on the mechanism of this phenomenon showed that the proteins stabilize the dimeric structure of TbTIM, and that this is the driving force that promotes the formation of active dimers. Similar data were obtained with TIM from three other species. The heat changes that occur when TbTIM is mixed with lysozyme were determined by isothermal titration calorimetry; the results provided direct evidence of the weak interaction between apparently unrelated proteins. The data, therefore, are strongly suggestive that the numerous protein-protein interactions that occur in the intracellular space are an additional control factor in the formation and stability of proteins.

## Introduction

The mechanisms that lead to the formation of the three-dimensional functionally active structure of proteins are being extensively investigated. Studies are being carried out with proteins that normally exist as monomers, as well as in proteins formed by various subunits. In the case of the latter proteins, there are the additional questions of how monomers interact and how the oligomeric state is stabilized [1]. In addition to identification of the intrinsic factors that lead to the formation of native proteins, there are also questions on whether the events that are observed *in vitro* reflect the processes that occur *in vivo*. As detailed in several reviews, significant advances have been made along this line [1]–[3]. An additional problem that has been in the mind of many investigators is that the test tube conditions in which the enzymes are traditionally studied differ dramatically from those that operate in the intracellular milieu. In this regard, a particularly relevant point is that within the cell the concentration of protein may be as high 400 mg per ml [4]–[8], a situation that has been termed crowding. The effect of crowding on the biochemistry and physiology of the cell has been mainly studied from the point of view of excluded volume, the fraction of the total volume that is unavailable to other molecules, and how it affects the rates and equilibria of biochemical reactions [4]–[8]. Aside from the effect of excluded volume on the energetics and kinetics of intracellular reactions, it could be hypothesized that in a crowded milieu, there would be many interactions between unrelated proteins. If such interactions are indeed occurring in the intracellular space, it follows that they will necessarily have a profound effect on the formation and stability of native proteins. Indeed, in an instructive article, Schellman [9] made a quantitative evaluation of Le Chatelier's principle that indicates that for example, the binding of ligands to a dimer will shift the equilibrium of the association reaction toward the dimer; conversely, ligand binding to one of the intermediate species in dimer formation will affect adversely the formation of the dimer. On these grounds, we explored if a model protein has the capacity to interact with other apparently unrelated proteins. To this end, we determined the effect of eight different proteins on the reactivation of homodimeric triosephosphate isomerase (TIM) from guanidinium chloride (GdnHCl) unfolded monomers, and on the stability of the dimer.

The proteins chosen differ markedly in molecular weight and isolectric point and were arbitrarily considered as representatives of intracellular proteins. We chose TIM because there is extensive knowledge of the kinetics and energetics of the route of active dimer formation from GdnHCl or urea unfolded monomers [10]–[14]. There is also detailed knowledge of the crystal structure of TIM from 18 different species at good resolution levels. All TIMs belong to the family of α/β barrel structures; the center of the barrel is formed by 8 β-strands which are surrounded by 8 α-helices; the helices and the strands are joined by loops. The kinetics of the catalytic activity of TIM [15], [16] and the dynamics of the enzyme in the resting and active state have been thoroughly investigated [17], [18].

We found that the eight proteins induce a several-fold increase in the extent to which TbTIM is reactivated. Further studies showed that this was due to a protein induced stabilization of the TIM dimer. Similar data were obtained when the effect of bovine serum albumin (BSA) was assessed in TIMs from three other species. Isothermal titration calorimetry provided direct evidence of the binding of lysozyme to TIM. Thus, the data indicate that in addition to a diminution in excluded volume, the interactions that may occur between unrelated proteins in the crowded intracellular space influence the formation and stability of native proteins.

## Results

The reactivation of TIM from GdnHCl unfolded monomers has been extensively investigated. Many of the studies have been carried out by dilution of the mixture in which the enzyme was denatured followed by measurements of activity. The results of various groups [11], [12], [14] show that TIM reactivation involves the following reaction sequence(1)
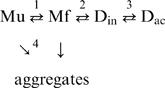
In reaction 1, unfolded monomers (M_u_) are transformed into folded monomers (M_f_). In reaction 2 the folded monomers associate and form inactive dimers (D_in_); through reaction 3, the inactive dimers undergo internal structural arrangements and form active dimers (D_ac_). Since reaction 2 is bimolecular, its rate depends on protein concentration; the rest of the reactions are uni-molecular and independent of protein concentration. A common observation in reactivation studies is that not all the protein that is allowed to undergo reactivation ends up as active dimers. In such cases, it is assumed that the missing fraction corresponds to incorrectly folded monomers or aggregates that are formed from either unfolded or folded monomers (reaction 4 in the scheme).

**Figure 1:**
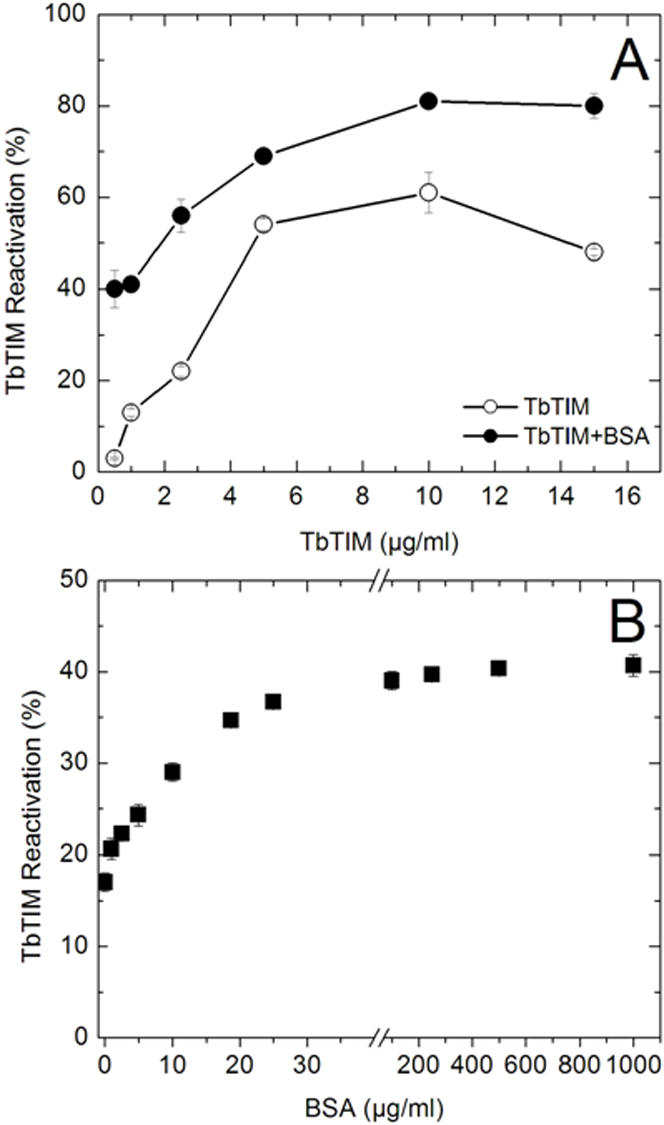
Reactivation of TbTIM with and without BSA. In A, denatured TbTIM was allowed to reactivate in 100 mM triethanolamine, 10 mM EDTA and 200 mM GdnHCl (pH 7.4) that contained the indicated concentrations of the enzyme without and with 19 µg BSA/ml (open and closed circles, respectively). In B, the reactivation media contained 1 µg/ml TbTIM and the indicated concentrations of BSA. After 24 hours at 25°C, activity was determined. The ordinate shows the extent of reactivation; 100% is the activity of the starting TbTIM which was 3353 and 3488 µmol/min/mg in experiments A and B, respectively.

Denatured TbTIM was allowed to reactivate at protein concentrations that ranged from 0.25 to 15 µg/ml without and with BSA in the reactivation media. The data in Figure 1A show the activity that was reached after 24 hours of reactivation. At this time the activity had remained constant for several hours (see below); thus, the results indicate the maximal extent of reactivation. In the absence of BSA, and in consonance with other works [11], [12], [14], we observed that the extent of reactivation increased with protein concentration, indicating that the rate of reaction 2 is central in the reactivation of TbTIM. Note that in all cases, especially at concentrations lower than 2.5 µg/ml, there was a substantial amount of enzyme that did not appear as active dimers. Figure 1A also shows that at all TbTIM concentrations, BSA increased the magnitude of active dimer formation. Relative to the respective controls, the number of times that BSA increased the formation of active dimers was higher at the lower protein concentrations. Thus, in the experiments that follow TbTIM was used at a concentration of 1 µg/ml.

There are many reports in which the reactivation of various enzymes has been measured in presence of BSA [for example see 19]–[23]. With the exception of citrate synthase, in which a favorable effect of BSA on reactivation was observed at concentrations of micrograms per ml [21], in most of the studies the action of BSA has been studied at concentrations of several, or even hundreds of mg per ml. Therefore, it is rather remarkable that the titration curves of different concentrations of BSA on TbTIM reactivation (Fig. 1B) showed that with 1 µg of TbTIM per/ml (36 nM monomer concentration), the near-maximal favorable effect of BSA was attained at concentrations of about 19 µg/ml (300 nM).

### Time course of TbTIM reactivation and the effect of BSA

**Figure 2:**
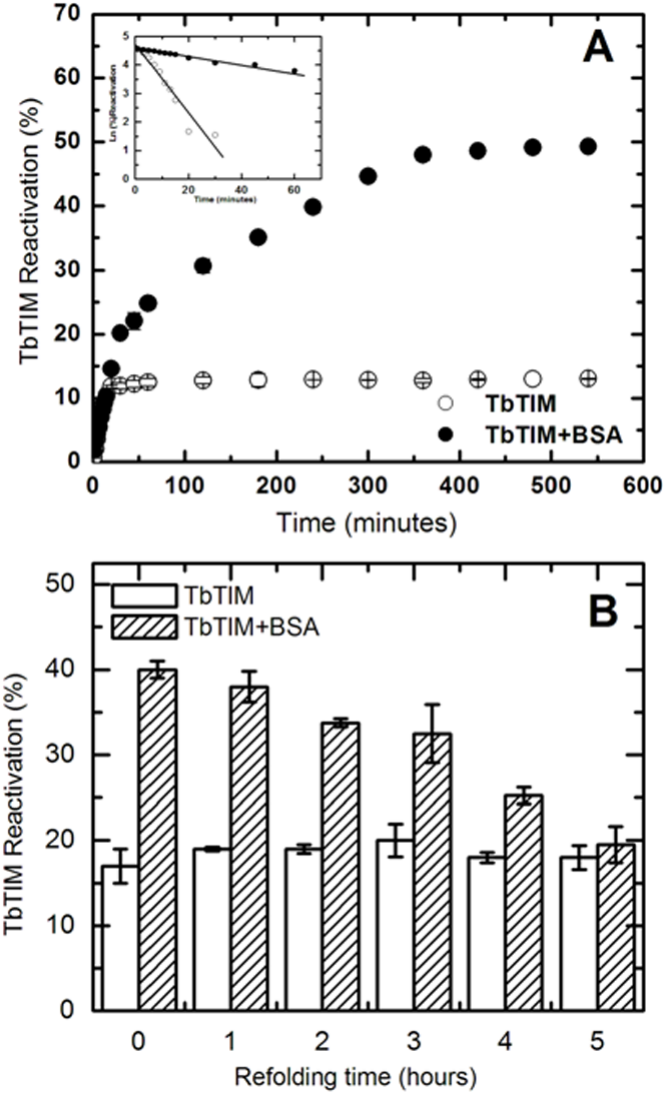
Time course of TbTIM reactivation . The conditions were as in Figure 1. The concentration of TbTIM in the reactivation buffer was 1 µg/ml and that of BSA 19 µg/ml. In A, the appearance of activity is plotted against time; 100% is the activity of native TbTIM, 3391 µmol/min/mg. The inset shows the ln of the appearance of activity versus time, where ln = [(Maximal activity reached−activity at time x)/maximal activity]×100. B shows the effect of adding BSA (19 µg/ml) after the enzyme was allowed to reactivate for 1, 2, 3, 4, and 5 hours; after 24 hours, activity was determined. In the zero time sample, denatured TbTIM was added to media that contained BSA. The activity of native TbTIM was 3121 µmol/min/mg; this was considered 100%.

In the absence of BSA, the time course of reactivation of unfolded monomers at a concentration of 1 µg/ml showed a progressive increase in activity for about 30 minutes (Fig. 2A). Afterwards activity remained constant for several hours, albeit it is noted that after 24 hours, we frequently observed a 5–10% decrease, relative to the observed after 30 min of reactivation. In presence of BSA, the time course of reactivation was strikingly different. After dilution (time zero in Figure 2A), the progressive increase in activity for the first 20 min was nearly the same with and without BSA. However, at longer reactivation times, when the activity of the control had reached a nearly constant level, the activity in presence of BSA continued to increase; it was only after approximately 6 hours that the activity reached a maximal constant level. Using the point of maximal activity with and without BSA as 100%, we calculated the first order rate constant by plotting the ln of the appearance of activity against time. In the two conditions, the two curves yielded straight lines (inset, Fig. 2A), albeit in presence of BSA the pseudo-first order rate constant was about seven times lower (0.128 min^−1^ in the control and 0.017 min^−1^ in presence of BSA).

The finding that BSA increased the formation of active dimers at times in which the reactivation of the control was at a constant level, prompted us to explore if BSA increases the formation of active dimers after the enzyme had been allowed to reactivate for substantial lengths of time. Figure 2B shows the effect of adding BSA to TbTIM that had been allowed to reactivate for 1, 2, 3, 4, and 5 hours; as a point of reference, the effect of BSA added at time zero is also shown. BSA added to the enzyme that had been incubated in the reactivation media for 1 hour induced an increase in activity comparable to that observed when BSA was introduced at time zero. Since the transition of unfolded to folded monomers takes place within one minute [14], the data of Fig. 2B show that the favorable effect of BSA is not on monomer folding.

BSA introduced at time zero and after one hour of reactivation induced similar effects (Fig. 2B); however, its favorable action diminished when it was added at progressively longer times. Indeed, when BSA was added after the enzyme had been standing for 5 hours in the reactivation media, its effect was rather modest. We would like to emphasize that the change in the sensitivity to BSA occurred in the times in which the amount of active dimers and incorrect structures were at a constant level (see Fig. 2A). This is of importance, since it indicates that in the state in which the amount of active dimers is constant, there are two types of incorrect structures: those that may be transformed into active dimers through the action of BSA, and those that are not. In all likelihood, the BSA insensitive fraction corresponds to aggregates. In fact, we observed that BSA did not induce the appearance of activity in aggregates that were prepared by dialysis of a mixture that contained 6 M GdnHCl and 1 mg/ml of TbTIM. The transition of the BSA-sensitive to the BSA-insensitive protein is rather slow. From the data in Figure 2B, it may be calculated that the half-time for the transition of BSA sensitive to BSA insensitive incorrect structures is around 2.5 hours.

### Effect of BSA on the stability of TIM dimers

The question that arises from the preceding observations concerns the mechanism through which BSA increases the formation of active dimers. In this context, please note in scheme 1 that in the state in which the activity of reactivated enzymes is constant, the existent dimers are in equilibrium with folded monomers (steps 2 and 3 in reaction sequence I). Therefore, we reasoned that the favorable effect of BSA could be due to a shift of the equilibrium between folded monomers and dimers toward the latter species by stabilization of the dimers. This possibility was explored by measuring the specific activity of TIM after it was incubated at various concentrations with and without BSA. The rationale of the experiments was that the incubation of TIM dimers at concentrations below the association constant of the monomers brings about an increase in the population of monomers, and because TIM monomers are inactive, there would be a decrease in the *specific activity* of the enzyme. Thus, if BSA increases the stability of the dimers, it would be expected that in presence of BSA the population of dimers at low protein concentrations would be higher than in its absence

**Figure 3:**
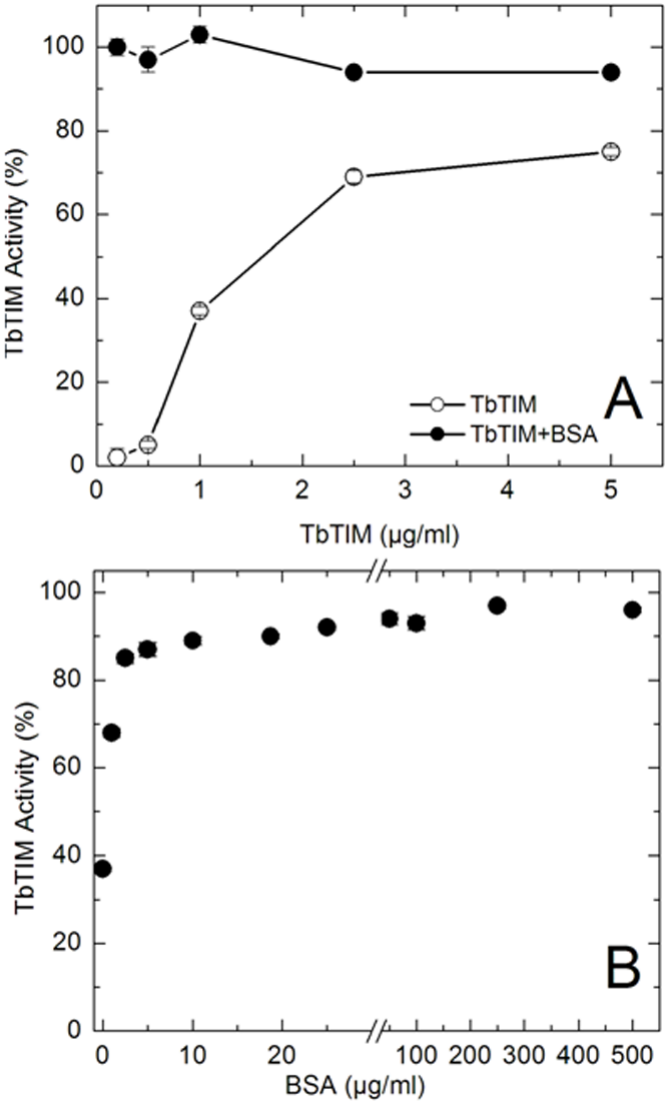
Effect of BSA on the stability of TbTIM dimers. In A, the indicated concentrations of native TbTIM dimers were incubated with and without 19 µg/ml BSA; after 24 hours activity was recorded. 100% is the activity at time zero (3401 µmol/min/mg). Note that with 5 µg/ml TbTIM, activity dropped by about 20%; at a concentration of 10 µg/ml, the activity remained unchanged (not shown). In B, 1 µg/ml TbTIM was incubated with the indicated BSA concentrations for 24 hours.

The results showed that in the absence of BSA, the specific activity of TbTIM decreased as its concentration was progressively diminished (Fig. 3A); this reflects the dissociation of the dimer as the concentration of protein is gradually diminished. From the data, we calculated that the concentration in which the specific activity of the enzyme dropped by 50% was 0.018 nM dimer concentration (1 µg protein/ml). In presence of BSA the results were markedly different; the enzyme expressed the same high specific activity at all the concentrations that were studied (Fig. 3A). Therefore, these data evince that BSA exerts a strong stabilizing effect of the TIM dimer. In this regard, it is relevant that the concentration of BSA that induces stabilization of the TbTIM dimer is in the same range as that which induces enhancement of active dimer formation (compare data in Fig. 1B and Fig. 3B).

### The effect of different proteins in active dimer formation and dimer stability

**Figure 4:**
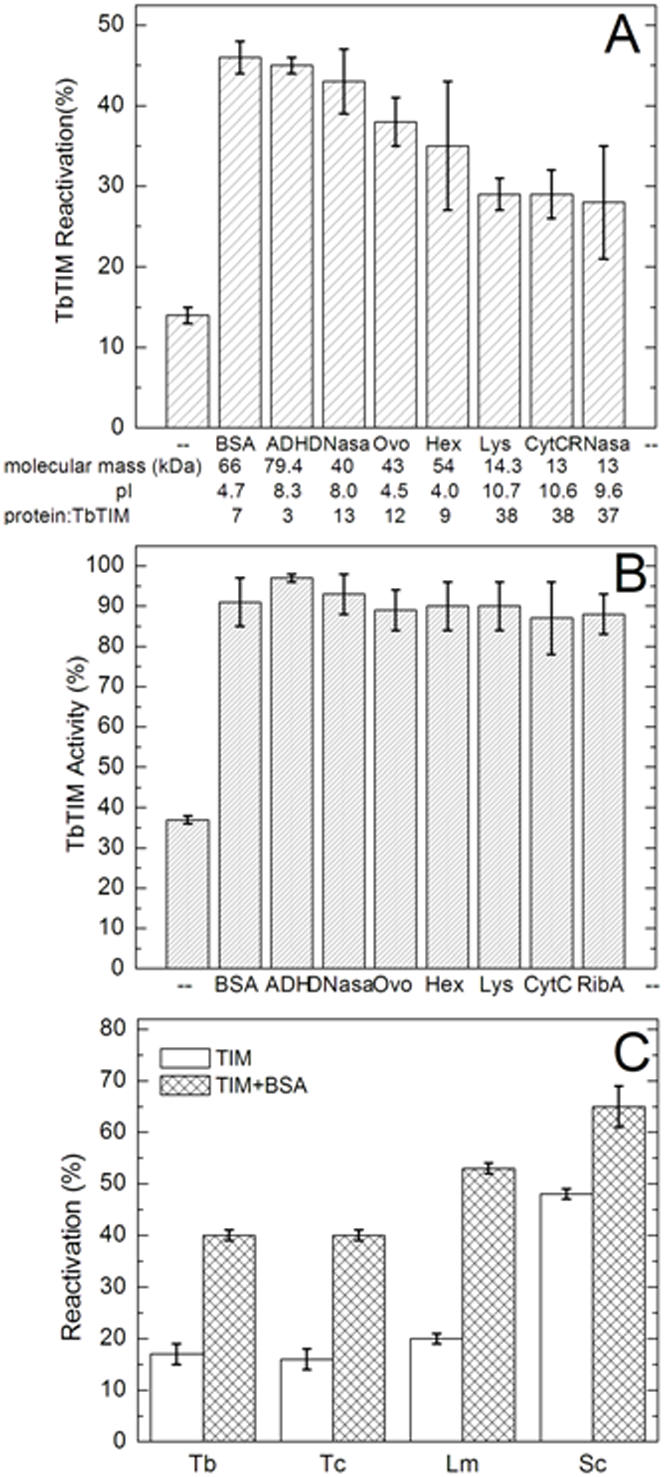
Reactivation and stability of TbTIM with various proteins, and reactivation of different TIMs. In A, 1 µg/ml of TbTIM was allowed to reactivate in presence of 19 µg/ml of the indicated proteins for 24 hours. The bottom of Figure A shows the molecular weights, isoelectric points and the molar ratio of protein:TbTIM of BSA; ADH: alcohol dehydrogenase; DNasa: deoxyribonuclease; Ovo: ovalbumin; Hex: hexokinase; Lyso: lysozyme; CytC: cytocrome c; RNasa: ribonuclease A. B shows the effect of 19 µg/ml of the same proteins on the stability of 1 µg/ml TbTIM dimers after 24 hours of incubation. Note that in the absence of the proteins, TbTIM lost 64% of its activity; in the presence of the proteins, hardly any activity was lost. The activity of native TbTIM was 3322 µmol/min/mg (100%). In C, the effect of 19 µg/ml BSA on the reactivation of 1 µg/ml of TIM from *T. cruzi* (Tc) *Leishmania mexicana* (Lm) and *Saccharomyces cerevisiae* (Sc) is shown. Data with TbTIM are included. The reactivation time was 24 hours. The activities of native TbTIM, TcTIM, LmTIM, and ScTIM were 3422, 3638, 3762, and 7572 µmol/min/mg, respectively. The respective activities were considered as 100%.

To probe the specificity of BSA action, we determined the effect of alcohol dehydrogenase, deoxyribonuclease, ribonuclease, lysozyme, ovalbumin, cytochrome c and hexokinase at a concentration of 19 µg/ml on the reactivation of TbTIM and stability of the dimer. All the proteins studied increased the formation of active dimers (Fig. 4A). Although there were differences on the magnitude of their favorable effect, there was no apparent correlation between the isoelectric point of the proteins (Figure 4) and their effect on TbTIM reactivation. For example, BSA and alcohol dehydrogenase induce similar effects, albeit there is a difference of 4 pH units in their isoelectric point. On the other hand, the data showed that on a molar ratio (protein:TbTIM), the larger proteins were more effective than the small proteins, suggesting that the surface area of the protein is related to the favorable effect of the proteins.

We also determined if the various proteins confer stability to TbTIM dimers. For these experiments we incubated TbTIM at a concentration of 1 µg/ml; at this concentration the specific activity of the enzyme dropped by about 60% in 24 hours (Fig. 4B). In presence of the tested proteins, the activity of the enzyme remained unchanged, indicating that in the presence of the proteins the dimer is stable.

### Effect of BSA on the reactivation of TIM from various sources

In order to ascertain if the effect of BSA was specific for TbTIM, we explored its effect on the reactivation of TIM from *T. cruzi, Leishmania mexicana*, and *Saccharomyces cerevisiae*. The protocols were identical to those used with TbTIM. The various denatured TIMs were allowed to reactivate at a concentration of 1 µg/ml of TIM protein without and with 19 µg/ml of BSA. In all TIMs, BSA promoted the formation of active dimers (Fig. 4C). Thus, the action of BSA is not limited to TbTIM.

### Calorimetric changes in the binding of TbTIM to lysozyme

**Figure 5:**
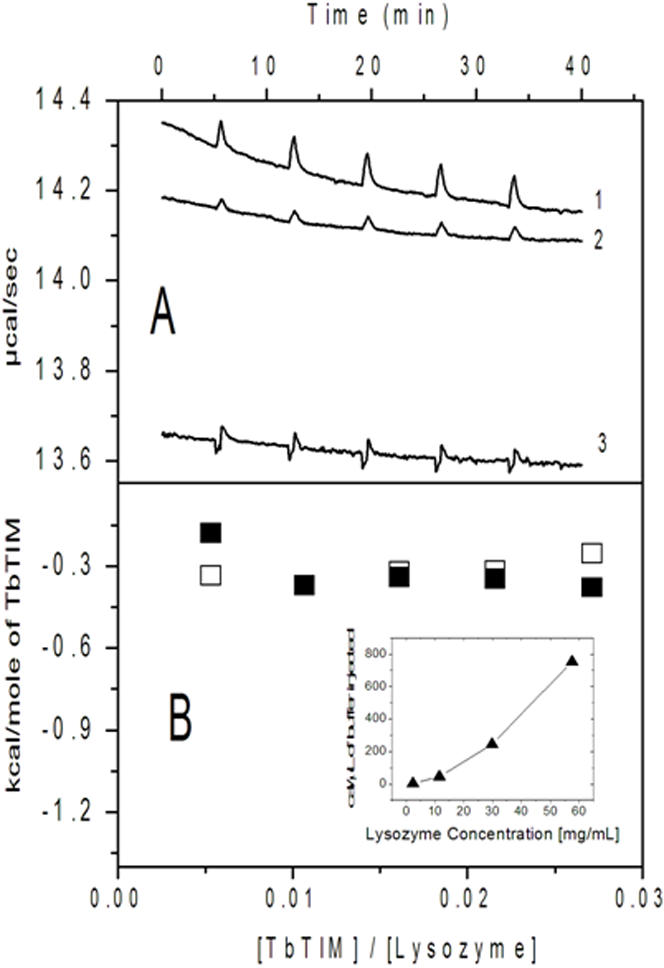
Heat changes of the interaction of TbTIM with lysozyme. Panel A shows (1) the ITC heat signals observed when 15 µl of buffer were repeatedly injected into a solution of 10 mg/ml lysozyme. (2) The heat changes that occurred when 15 µl of a solution of 17 mg TbTIM per ml were injected into a solution of 10 mg/ml lysozyme. (3) The calorimetric response of repeated injections of a TbTIM solution into buffer alone. Panel B depicts the heat evolved in the interaction between TbTIM and lysozyme as a function of the TbTIM/lysozyme molar ratio. Open squares are heats obtained from the calorimetric traces in A; closed squares are a replica. The average enthalpy change was −0.32 kcal/mole of TbTIM. Inset: heat due to the dissociation of the self-associated lysozyme as function of lysozyme concentration in the main cell.

We used isothermal titration calorimetry to gain insight into the energy changes that occur when TbTIM is mixed with one the proteins that induce dimer stabilization. As noted in the Methods section, these measurements were made with TbTIM and lysozyme after we established the conditions that yielded reliable data. For the experiments, high concentrations of protein were introduced into both, the main cell and the syringe. Under these conditions, the injection buffer alone into the lysozyme solution induced a relatively large endothermic signal. Accordingly, we examined the heat changes that occur when 15 µl of buffer was injected into cells that contained different lysozyme concentrations (inset Fig. 5); the aim was to find a condition in which the signal induced by TbTIM was distinguishable from that induced by buffer alone. Good results were obtained with 10 mg lysozyme per ml 0.7 mM) in the main cell and 0.35 mM TbTIM in the syringe. The results of repeated injections of 15 µl of buffer alone or 15 µl of 0.35 mM TbTIM into 1.5 ml of 0.7 mM lysozyme, and the heat of dilution of TbTIM injected into buffer alone are shown in Figure 5A. The heat change induced by the injection of buffer into the lysozyme solution and that induced by the injection of TbTIM into buffer were subtracted from the experimental (TbTIM into lysozyme); the data are in Fig. 5B. The value was divided by the molar amount of TbTIM injected in order to obtain the binding enthalpy that was −0.32 kcal/mol.

## Discussion

We have found that eight different unrelated proteins shift the unfolded monomer-dimer equilibrium of TIM toward the latter species. A relevant feature of the action of the tested proteins is that they induce maximal enhancement of dimer formation at concentrations of micrograms per ml. This indicates that their action is not due to a crowding effect, since concentrations of hundreds of mg/ml are needed to mimic the conditions of the intracellular milieu. Indeed, it has been put forth that at concentrations of 1 to 10 mg of protein per ml, crowding is negligible [5]. Although the findings indicate that the favorable effect of proteins on the formation and stability is not due to a crowding effect, it is evident that the crowded milieu of the intracellular space is propitious for the interaction between many different proteins. Therefore, it is very likely that the phenomenon we have observed takes place in the interior of cells.

In this regard, and because in the interior of cells the formation of the many native proteins depends on the existence of chaperones, it could be considered that the proteins we studied favor dimer formation through a chaperone-like effect. This possibility is relevant, particularly because we observed that during reactivation, the partition of proteins into nonproductive species is importantly diminished. However, the action of proteins here described is ATP independent, whereas the action of chaperones on protein formation involves the binding and release of intermediates of the folding pathway through a process that is linked to ATP binding, hydrolysis, and product release [24]–[26]. Moreover, our data indicate that the various proteins favor TIM reactivation through an entirely different mechanism. In fact, our overall results show that the eight proteins stabilize the dimeric structure of TIMs, and that this is the driving force for the favorable action of proteins on the formation of active dimers from unfolded monomers.

The measurements of the heat changes that occur when lysozyme is mixed with TbTIM provided direct evidence of interaction; nonetheless it is important to note that energetically the interactions are quite weak. Thus, in both, specificity and energetics, the protein interactions here described are clearly distinct from those that operate for example, in the binding of substrates to enzymes, ligands and receptors, or protein-protein interaction involved in signal transduction. Indeed, we found that proteins of widely different molecular weights, isoelectric points, and aggregation states interact with TIMs from different sources. Thus, the interactions may be considered as *nonspecific*. It is noted however, that the present data also show that in the reactivation pathway (Reaction scheme 1), the proteins that were tested did not interact with TIM monomers, otherwise the formation of active dimers would have diminished or prevented. Instead, the data showed that the dimer was the sole or predominant species with which the proteins interact. Thus, the preference for a particular protein conformation indicates that the interactions exhibit a certain level of specificity. This is also in contrast with chaperones that only recognize intermediates of the folding pathway.

Taken together, the results of this work indicate that TIM dimers have the capacity to interact with a wide variety of proteins. This would be in consonance with reports that show that TIM interacts with actin, microtubules and membranes [27]–[30] and that TIM binding to the latter structures is affected in human TIM deficiencies [29]. Likewise, it has been shown [31] that TIM, as well as other glycolytic enzymes, interacts with the K_ATP_ channel of the plasma membrane and thereby regulates its function. Finally, it is noteworthy that Walden *et al*. [32]–[33] reported that TIM from the hyperthermophile *Thermoproteus tenax* exists in equilibrium between dimers and tetramers and that glycerol 1-phosphate dehydrogenase shifts the equilibrium toward de tetrameric form; it was also shown that glycerol 1-phosphate dehydrogenase protects the enzyme against heat inactivation [33]. Thus, the collective data indicate that TIM is rather promiscuous in so far that it interacts with several intracellular components.

The intracellular milieu in which proteins abound would seem to be an ideal system for the occurrence of interactions between unrelated proteins. On thermodynamic grounds [9], the interactions between native proteins or other intracellular components, would certainly be a driving force for shifting the equilibrium of the reactivation route towards the native protein; furthermore the interactions would also have a strong bearing on the stability of the native structure. In this regard, it is noted that the existence of interactions of proteins with other proteins and other intracellular structures has been known for a long time [for an illustrative and historical review, see ref. 34]. Now it is well established that many of the interactions increase the efficiency of metabolic pathways [35]–[36]. However, in the light of the present findings, it would seem that in the intracellular space, the interactions between unrelated proteins could have additional functions. Indeed, it could be that these weak interactions contribute importantly to the formation and stability of enzymes in the intracellular space.

## Materials and Methods

### Enzymes

The expression and purification of TbTIM and triosephosphate isomerases from *Trypanosoma cruzi* (TcTIM), *Leishmania mexicana* (LmTIM) and *Saccharomyces cerevisiae* (ScTIM) were performed as described in references [37]–[40 respectively]. The purified enzymes were maintained as a suspension in 100 mM triethanolamine, 10 mM EDTA, 1 mM dithiothreitol, and 75% saturated ammonium sulfate (pH 7.4) at 4°C. For the experiments, the suspensions were centrifuged and the pellets dissolved in 100 mM triethanolamine and 10 mM EDTA (pH 7.4) and dialyzed against the same buffer.

### Proteins

The following proteins were obtained from Sigma: fatty acid free BSA prepared from fraction V albumin; grade I lysozyme from chicken egg white; ethanol free alcohol dehydrogenase from equine liver; deoxyribonuclease II from bovine spleen; type VI ribonuclease A from bovine pancrease; type IV protease-free hexokinase from baker's yeast; type VII ovoalbumin; and cytochrome C from horse heart.

### Activity Assays

Activity in the direction of glyceraldehyde 3-phosphate to dihydroxyacetone phosphate was determined at 25°C as described elsewhere [38]. The reaction was started by the addition of TIM, generally 5 ng of protein. Activity was followed by the decrease in absorbance at 340 nm as a function of time in an HP8452 spectrophotometer with a multicell attachment.

### Denaturation and reactivation of the enzymes

The enzymes were denatured by incubation of 500 µg/mL for 1 hour in 100 mM triethanolamine, 10 mM EDTA, and 6 M GdnHCl (pH 7.4). Reactivation of the denatured TIM was initiated by diluting (at least 100-fold) the denaturated enzymes in media at pH 7.4 that contained 100 mM triethanolamine, 10 mM EDTA and 200 mM GdnHCl (final concentrations) and incubated at 25°C; where shown, the reactivation media also contained the proteins whose action on reactivation was studied. The final concentrations of denatured TbTIM and the proteins in the reactivation media are indicated in the Results section. Control experiments with native TbTIM showed that 200 mM GdnHCl did not change the activity and the structure of the enzyme. After adding the denatured enzyme to the reactivation media, aliquots of the mixture were withdrawn at the indicated times to measure activity. The incubations were carried out in Eppendorf tubes; it was checked that the proteins did not adsorbed to walls of the tubes by measuring the amount of protein after they were allowed to stand in the tubes for two hours.

### Stability of TIM dimers

Different concentrations of the native TIMs were incubated in 100 mM triethanolamine and 10 mM EDTA (pH 7.4), at 25°C for 24 hours, with and without the proteins whose effect was to be studied. After 24 hours of incubation at 25°C, aliquots were withdrawn to measure activity.

### Protein concentration

This was determined according to Pace *et al.* [41]. The concentration of the different TIMs was determined from their absorbance at 280 nm using the molecular extinction coefficients (є_280_) of 34950 M^−1^ cm^−1^ for TbTIM, 33460 M^−1^ cm^−1^ for TcTIM, 36440 M^−1^ cm^−1^ for LmTIM, 26664 M^−1^ cm^−1^ for ScTIM, and 42925 M^−1^ cm^−1^ for BSA. Solutions of the other proteins were made by weight.

### Isothermal Titration Calorimtetry (ITC)

The experiments were performed at 25°C in a high precision VP-ITC isothermal titration calorimeter (Microcal Inc.). As expected, the initial experiments showed that there was only a weak interaction between TbTIM and two of the proteins that we tested, namely BSA and lysozyme. We therefore performed several experiments in order to find the conditions in which reliable data could be obtained. The initial experiments showed that the main cell and the syringe had to contain high concentrations of protein, otherwise the heat changes were undetectable. However, at high concentrations and in consonance with previous reports [42]–[45], BSA and lysozyme underwent self-association. Thus, we had to find a protein concentration in which the heat of dilution did not mask the heat change that resulted from the interaction of the two proteins. In this regard, it is noted that the data with lysozyme were better than with BSA, hence, the experiments were made with lysozyme. With the other proteins, the cost of the experiments was prohibitive. The experimental conditions are detailed in the Results section. Prior to the experiments, TbTIM was dialyzed against 500 ml of the standard buffer; two changes were made. The last buffer was used to dissolve lysozyme. TbTIM and lysozyme solutions were passed through 0.22 µM Millipore filters prior to their transfer to the main cell or syringe.
